# Anthropometric status of 5–13-year-old children attending urban and rural primary schools in KwaZulu-Natal, South Africa

**DOI:** 10.3389/fpubh.2026.1803006

**Published:** 2026-05-15

**Authors:** Onwaba Makanjana, Ashika Naicker, Imana Pal

**Affiliations:** Department of Consumer Sciences Food and Nutrition, Durban University of Technology, Durban, South Africa

**Keywords:** anthropometric status of children, malnutrition, obesity, school-age children, stunting

## Abstract

**Background:**

The United Nations Sustainable Development Goal (SDG) 2 aims to end hunger and all forms of malnutrition by 2030. There is a high prevalence of malnutrition among school-age children in South Africa. This article seeks to determine the anthropometric status of learners between 5 and 13 years across quintile 1–5 primary schools in the iLembe district in KwaZulu-Natal in South Africa.

**Methods:**

This was a cross-sectional study. A calibrated electronic SECA scale and stadiometer was used to assess the weight and height of the participants, respectively. Data was analyzed using the World Health Organization Anthro Plus software, version 1.0.4. Correlations were conducted using SPSS® version 31.

**Results:**

Stunting was prevalent in all three schools. Among the 5–9-year-old group, low height for age was observed in 65.9% of boys and 7.1% of girls in school 1 (quintile 3). With all the schools, age groups and genders combined, the overall prevalence of overweight was 24.6%, with 12.0% of the participants being obese and 3.8% being severely obese.

**Conclusion:**

Findings showed a double burden of malnutrition among primary school children in KwaZulu-Natal irrespective of school quintile, therefore indicating a necessity to establish a healthy school food environment that improves the availability of healthy foods, and encourages physical activity to enhance child health outcomes.

## 1 Introduction

The United Nations Sustainable Development Goal (SDG) 2 aims to end hunger and all forms of malnutrition by 2030. Many countries are lagging in meeting this target. Global trends show that even though there has been a decrease in undernutrition, malnutrition is still prevalent as overnutrition has increased in various regions (1). Globally, approximately 2.4 billion people were moderately or severely food insecure in 2022. Food insecurity contributes to the high prevalence of malnutrition across countries (2). Despite the global targets of achieving a 40% reduction in stunting by 2025 (3), 148.1 million children were stunted in 2022. Forty-five and 37 million children were wasted and overweight, respectively. Globally, stunting and wasting were reported to be higher in rural areas compared to urban areas, and overweight is higher among children residing in urban areas (2).

The South African National Food and Nutrition Security Survey Report highlighted a high prevalence of malnutrition across age groups in South Africa. In children below 5 years, 28.8% were stunted, with 14.0 being moderately stunted and 14.8% being severely stunted. The overall prevalence of wasting was 5.3%, with 3.2% of the children being severely wasted. The report also showed that 7.7% of the children were underweight, with 3.7% being severely underweight and 3.9% being moderately underweight. Overweight was also prevalent among 22.6% of the children. This survey did not report on children between 6 years and 17 years (4).

South Africa is one of the countries with high levels of inequality (5). As one of the strategies to address inequalities in the schooling system, public schools in South Africa have been divided into five quintiles, with quintile 1 schools being the poorest and quintile 5 being the least poor schools. Quintile one to three schools have been classified as no-fee paying schools, whereas quintile four to five schools are fee-paying schools (6). In South Africa, malnutrition affects children in urban and rural areas. A study on the nutritional status of children between 6 and 15 years in a rural setting in South Africa found that 22% of the children were stunted, 5% of the children were severely stunted, and 27% of the children were underweight. Of the 27% of the children who were underweight, 6% were severely underweight. Furthermore, 43.4% of children who participated in this study had a combination of underweight and stunting (7). Similar findings were reported by Meko et al. (8), who reported stunting, wasting and overweight among school-age children aged 13 to 15 years in a study conducted in Bloemfontein in South Africa. The authors found that 27.5% of the children who participated in the study were stunted, and 10.3% of boys were severely stunted, compared to 4.2% of girls. Stunting was significantly higher among children in low and medium socio-economic groups compared to children from higher socio-economic groups. Wasting was found in 14.9% of the study participants, while 6% were overweight/obese. The risk of being overweight was higher at 15.6% (8).

The World Health Organization (WHO) identified factors that play a role in the occurrence of stunting in children. Community and societal factors such as food prices, trade policy, income, employment and livelihoods contribute to stunting. Access to healthcare, quality education and availability of micronutrient-rich foods also play a role. In the household environment, poor-quality foods with low micronutrient quality and low diversity, inadequate breastfeeding, insufficient care giving and re-current infections contribute to stunting. Many households in low- and middle-income countries have inadequate sanitation and water supply. This exposes children to recurrent infections, which may lead to reduced appetite and decreased food intake. Low levels of caregiver education, low social status of women and food insecurity also contribute to stunting in children. Maternal factors that cause stunting include adolescent pregnancy, poor nutrition prior to conception, during pregnancy and lactation and short maternal stature (9).

### 1.1 Rationale

Most studies assessing children’s nutritional status have focused on those under 5 years, leaving a research gap for school-aged children aged 5–13. This article seeks to determine the anthropometric status of learners between 5 and 13 years across quintile 1–5 primary schools in the iLembe district in KwaZulu-Natal (KZN). Stunting and wasting in school-age children negatively affects academic performance (10), and overweight and obesity negatively impacts the quality of life and well-being and are associated with a higher risk of developing non-communicable diseases later in life (11) and a high economic impact (12). By determining the anthropometric status of children in this age group and providing a comparison between the nutritional status of children attending schools in lower and higher quintiles, this study will inform the planning of contextualized interventions aimed at improving the health of school-age children. This study reports on one component of the larger research study that implemented the ‘Blueprint for improving the school environment’ project conducted by UNICEF in collaboration with a University in Durban, South Africa (13).

## 2 Materials and methods

### 2.1 Study design

This was a cross-sectional study with quantitative data obtained using anthropometric measurements.

### 2.2 Study setting

The study was conducted in three public primary schools in the iLembe district in KZN. The first school (school 1) was a quintile three (Q3) school with approximately 1574 learners, located in an urban area. The second school (school 2) was a quintile one (Q1) school located in a rural area, with approximately 200 learners. Both these schools are classified as no-fee paying schools and therefore rely on funding from the government. The third school (school 3) was a quintile five (Q5) school located in an urban area, with approximately 909 learners. The third school is a fee-paying school. Fee-paying schools receive less government funding (compared to non-fee-paying schools) and charge school fees from the learners as determined by the school governing body (13).

### 2.3 Study participants and sampling

The study participants were 183 children (65 boys and 118 girls) aged 5–13 years, attending grades R to 7 (kindergarten to second year of middle school). The participants were divided into two groups, namely, 5–9 years (*n* = 81) and 10–13 years (*n* = 102). A non-probability, voluntary response sampling approach was used. Class lists were obtained from each school, and information letters and consent forms were distributed to all eligible learners within the selected grades. Participation was based on parental consent. Only learners whose parents/guardians returned signed consent forms were included in the study. The recruitment process was coordinated by the research team in collaboration with school staff. To ensure consistency across the three schools, a standardised recruitment procedure was followed, including uniform distribution of study information, consent forms, and clear instructions to parents/guardians. The participating schools were preselected by the KZN Department of Education as pilot schools in the Blueprint for Improving the School Food Environment project.

### 2.4 Data collection and analysis

Data collection was conducted between September and November 2024. A calibrated electronic SECA scale was used to assess participants’ weight, and a SECA stadiometer was used to assess their height. Height measurements were rounded off to the nearest 0.1 cm and weight to the nearest 0.1 kg. The Mid-Upper Arm Circumference (MUAC) was measured utilizing a stretchable, flexible measuring tape to the nearest 0.1 cm. Measurements were obtained on the left arm at the midpoint between the acromion process of the scapula and the olecranon process of the ulna. The mid-point was identified with the individual in an upright position, arm relaxed and hanging freely at the side. The measuring tape was positioned securely around the arm without constricting the soft tissue. All measurements were taken twice, and an average was recorded. All the anthropometric measurements were conducted according to WHO recommendations (14).

Data was analyzed using the World Health Organization Anthro Plus software, version 1.0.4. (15). The WHO cut-off points used for wasting were low weight for age below −2 standard deviations (SD). Weight for age is applicable to children between 5 and 9 years only, as the WHO indicates that there is no reference data for weight for age for children beyond 10 years, as this indicator does not differentiate between height and Body Mass Index (BMI) in older children experiencing a growth spurt associated with puberty (15). Stunting was classified as low height for age below −2 SD. Overweight was classified as BMI for age above >+1 SD, which is equivalent to BMI 25 kg/m^2^ at 19 years, and obesity classified as >+2 SD, equivalent to BMI 30 kg/m^2^ at 19 years. Underweight was classified as low BMI for age was below −2SD (15). Correlations were conducted using SPSS® version 31 (16).

### 2.5 Ethics

Ethical approval was obtained from the Institutional Research Ethics Committee (IREC 013/23). Gatekeeper permission was obtained from the Department of Basic Education. Informed consent was obtained from the school principals and from parents and/ guardians of the children. This study only included children whose parents/guardians’ consent was received.

## 3 Results

Of the total sample in the current study, 81 children (boys *n* = 28; girls *n* = 51) were between 5 and 9 years old. As reflected in Table 1, 5.9% of the boys in school 1 and 3.6% of boys in school 3 were underweight. Underweight was found in the two urban schools in quintiles 3 and 5, with an overall prevalence across gender and age groups combined of 3.2% in school 1 and 2.7% in school 3. None of the participants from the quintile 1 school (school 2) were underweight. Among the 5–9-year-olds from the three schools combined (*n* = 81), the overall prevalence of underweight was 2.5%.

**Table 1:** Underweight for 5–9-year-old children.

Schools	*n*	Severely underweight	Underweight	Not underweight	WAZ	
		% <−3SD	% <−2SD		Mean	SD
All schools combined (5–9-year-olds)	81	0	2.5	97.5	0.47	1.408
School 1 (Q3)						
Genders Combined	31	0	3.2	96.8	0.32	1.394
Boys	17	0	5.9	94.1	0.09	1.455
Girls	14	0	0	100	0.70	1.265
School 2 (Q1)						
Genders Combined	14	0	0	100	0.39	0.731
Boys	6	0	0	100	0.20	0.756
Girls	8	0	0	100	0.52	0.731
School 3 (Q5)						
Genders Combined	36	0	2.7	96.7	0.62	1.619
Girls	8	0	0	100	0.47	1.404
Boys	28	0	3.6	96.4	0.67	1.697

As reflected in Table 2, stunting was found in all three schools. Among the 5-9-year-age group, low height for age (<-2SD) was found in 5.9% of boys and 7.1% of girls in school 1 (quintile 3). In school 3 (quintile 5), 3.6% of girls between 5-9 years had a low height for age. In the 10-13-year-old age group, stunting was observed in schools 1 and 2. In school 1, 4.0% of boys in this age group were stunted, and none of the girls in this age group were stunted. School 2 (quintile 1) had a higher prevalence of stunting among boys, of which 33.3% of the boys were stunted. When gender and age groups were combined, the overall prevalence of stunting was 3.5% in school 1 (quintile 3), 4.0% in school 2, and 1.4% in school 3 (quintile 5).

**Table 2:** Stunting for all age groups.

Schools	*n*	Severely stunted	Stunted	Not stunted	HAZ	
		% <−3SD	% <−2SD		Mean	SD
All schools combined	183	0	2.7	97.3	−0.05	1.052
School 1 (Q3)	85	0	3.5	96.5	−0.18	1.129
5–9 years						
All	31	0	6.5	93.5	−0.49	0.917
Boys	17	0	5.9	94.1	−0.56	0.950
Girls	14	0	7.1	92.9	−0.40	0.903
10–13 years						
All	54	0	1.9	98.1	−0.04	1.207
Boys	24	0	4.0	96.0	0.17	1.453
Girls	30	0	0	100	−0.14	0.972
School 2 (Q1)	25	0	4.0	96.0	−0.16	0.933
5–9 years						
All	14	0	0	100	−0.18	0.804
Boys	6	0	0	100	−0.46	0.635
Girls	8	0	0	100	0.04	0.891
10–13 years						
All	11	0	9.1	90.9	−0.14	1.117
Boys	3	0	33.3	66.7	−0.45	1.711
Girls	8	0	0	100	−0.02	0.942
School 3 (Q5)	73	0	1.4	98.6	0.15	0.978
5–9 years						
All	37	0	2.7	97.3	0.04	1.060
Boys	9	0	0	100	−0.20	0.848
Girls	28	0	3.6	96.4	0.12	1.122
10–13 years						
All	36	0	0	100	0.26	0.887
Boys	6	0	0	100	0.28	0.978
Girls	30	0	0	100	0.26	0.886

Results in Table 3 show a high prevalence of overweight and obesity in all three schools, with schools 1 and 3 also having participants who were severely obese. With all the schools and age groups and genders combined, the overall prevalence of overweight was 24.6%, with 12.0% of the participants being obese and 3.8% being severely obese. In school 1 (quintile 3), 24.7% of the participants were overweight, 10.6% were obese, and 3.5% were severely obese. In school 2 (quintile 1), 24.0% of the participants were overweight, and 16.0% were obese. In school 3 (quintile 5), 24.7% of the participants were overweight, 12.3% were obese, and 5.5% were severely obese. Among boys in both age groups combined, 24.4% were overweight, 7.3% were obese, and 2.4% were severely obese in school 1. In school 2 (quintile 1), 11.1% of boys were overweight, and 22.2% were obese. In school 3 (quintile 5), 20.0% of the boys were obese and 6.7% were severely obese. Among the girls, schools 2 and 3 had higher levels of overweight compared to school 1 (quintile 3). In school 1, 25.0% of the girls were overweight, 13.6% were obese, and 4.5% were severely obese. In school 2 (quintile 1), 31.3% of the girls were overweight, and 12.5% were obese. In school 3 (quintile 5), 31.0% of the girls were overweight, 10.3% were obese, and 5.2% were severely obese.

**Table 3:** Overweight for all age groups.

Schools	*n*	Severely thin	Thin	Normal	Overweight	Obese	Severe obesity	BAZ	
		% <−3SD	% <−2SD		% >+1SD	% >+2SD	% +3SD	Mean	SD
All schools combined	183	0	1.1	58.5	24.6	12.0	3.8	0.69	1.366
School 1 (Q3)	85	0	2.4	58.8	24.7	10.6	3.5	0.62	1.305
Boys combined	41	0	0	65.9	24.4	7.3	2.4	0.43	1.218
5–9 years	17	0	0	64.7	23.5	5.9	5.9	0.47	1.397
10–13 years	24	0	0	66.7	25.0	8.3	0	0.41	1.104
Girls combined	44	0	4.5	52.3	25.0	13.6	4.5	0.80	1.370
5–9 years	14	0	0	35.7	42.9	14.3	7.1	1.18	1.058
10–13 years	30	0	6.7	60.0	16.7	13.3	3.3	0.63	1.477
School 2 (Q1)	25	0	0	60.0	24.0	16.0	0	0.77	0.945
Boys combined	9	0	0	66.7	11.1	22.2	0	0.70	0.981
5–9 years:	6	0	0	66.7	16.7	16.7	0	0.66	0.968
10–13 years	3	0	0	66.7	0	33.3	0	0.80	1.220
Girls combined	16	0	0	56.3	31.3	12.5	0	0.81	0.955
5–9 years	8	0	0	75.0	12.5	12.5	0	0.63	0.825
10–13 years	8	0	0	37.5	50.0	12.5	0	1.0	1.093
School 3 (Q5)	73	0	0	57.5	24.7	12.3	5.5	0.74	1.557
Boys combined	15	0	0	73.3	0	20.0	6.7	0.59	1.418
5–9 years	9	0	0	77.8	0	11.1	11.1	0.62	1.417
10–13 years	6	0	0	66.7	0	33.3	0	0.54	1.552
Girls combined	58	0	0	53.4	31.0	10.3	5.2	0.78	1.601
5–9 years	28	0	0	60.7	25.0	7.1	7.1	0.80	1.797
10–13 years	30	0	0	46.7	36.7	13.3	3.3	0.80	1.424

Using Fisher’s Exact test, there were no significant differences in weight-for-age (WAZ), height-for-age (HAZ), or BMI-for-age (BAZ) when comparing location, age groups, gender, or school quintiles (Table 4). The Kruskal–Wallis test showed that Mid-Upper Arm Circumference (MUAC) differs significantly across BMI categories, *χ*^2^ (3) = 42.167, *p* < 0.001. *Post hoc* analysis with Bonferroni adjustment using the Mann–Whitney test shows that MUAC was significantly lower for those with normal BMI than for those who are overweight (*p* < 0.001), obese (*p* < 0.001) and severely obese (*p* < 0.001); MUAC is significantly lower for those who are overweight than for those who are obese (*p* = 0.005) or severely obese (*p* < 0.001) (Table 4).

**Table 4:** Comparative analysis of anthropometric indicators across groups (*n* = 183).

Variable	Category	WAZ	HAZ	BAZ
Location	Urban (*n* = 25)	0.513	0.676	0.769
	Rural (*n* = 158)			
Age group	5–9 years (*n* = 82)	–	0.489	0.337
	10–13 years (*n* = 101)			
Gender	Boys (*n* = 65)	0.791	0.246	0.274
	Girls (*n* = 118)			
School quintile	Quintile 1 (*n* = 25)	0.802	0.649	0.828
	Quintile 3 (*n* = 85)			
	Quintile 5 (*n* = 73)			
MUAC vs. BMI	Across BMI categories (*n* = 82)	–	–	***p* < 0.001**

Bold value indicate statistical significance (*p* < 0.001).

**Figure 1:**
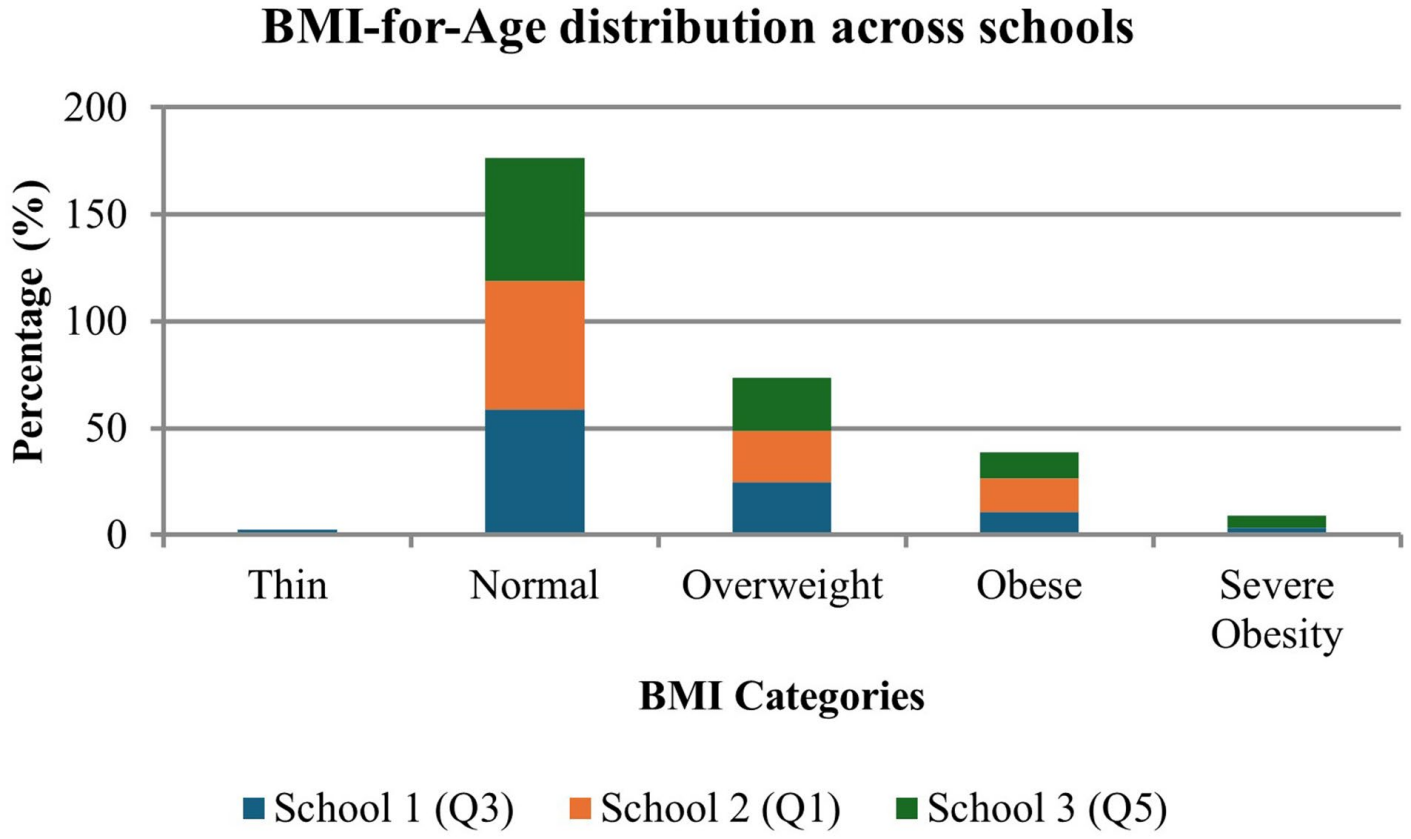
Distribution of BMI-for-Age (BAZ) categories across schools.

Figure 1 presents the distribution of BMI-for-Age (BAZ) categories across schools and quintiles. The normal BMI category dominates across all schools. Across all schools, the prevalence of overweight and obesity combined was 40.4%. The highest combined obesity and severely obese rate was in the quintile 5 school, which had the highest severe obesity prevalence (17.8%). There was no statistically significant difference in BMI distributions across schools (*p* = 0.828), but the suggest emerging overweight and obesity issues across all quintiles.

## 4 Discussion

This study presents anthropometric data on the nutritional status of school-going children aged 5 to 13 years in KZN, South Africa. Nutritional status is a key determinant of overall health and an indicator of the well-being of schoolchildren in a specific region (17). There is significant importance in highlighting the nutritional status of children in developing countries, not only to address the health of future generations but also to support wider regional development. The study’s results indicate the coexistence of undernutrition and overnutrition and focus on the double burden of malnutrition in South Africa.

### 4.1 Undernutrition among school-aged children

The study’s results indicate that the prevalence of underweight (2.5%) and stunting (2.7%) is lower than the national standard for the country (4). However, the incidence of stunting across schools with varying socio-economic profiles remains a significant concern. In all three schools (Q1, Q3, and Q5), stunting was present among learners, indicating that undernutrition affects households across socio-economic conditions. These findings support previous studies conducted in different rural and urban settings in South Africa, which documented cases of stunting among schoolchildren (7, 8).

The causes of stunting can be attributed to long-term nutritional deprivation, which often starts with the first 1000 days of life and can continue even when access to food is improved later in life (9). The incidence of underweight in rural Q1 schools is lower and may be credited to social protection schemes such as the National School Nutrition Program (NSNP), which provides at least two daily meal for learners in no-fee paying schools. School stakeholders regard the NSNP as an essential tool to help children in low-income communities who are experiencing short-term hunger by increasing their access to healthy food. However, some challenges, such as poor food quality, an unstandardised menu, and insufficient implementation, could limit program effectiveness (13). Stunting and other long-term nutritional consequences are often the result of malnutrition in childhood, and although the NSNP does improve dietary intake and school attendance, systematic evidence suggests its effects on these outcomes are less consistent (18). Thus, although the NSNP benefits from short-term malnutrition, it fails to accomplish much to help with long-term issues like stunting. This result emphasizes the need for improved program quality, strong oversight, and integration with more comprehensive nutrition-sensitive interventions.

Consistent with findings from other studies in South Africa and across Africa, this study also identified a higher incidence of stunting among boys (8). Factors including biological vulnerability, increased energy requirements, and gender-based disparities in caregiving and feeding practices may contribute to these observed differences.

### 4.2 Overweight and obesity across all quintiles

This study’s significant finding is the high combined prevalence of overweight and obesity (40.4%) across all schools, regardless of quintile level. This rate exceeds national projections for younger children and is in line with worldwide data showing rising rates of childhood obesity and overweight in low- and middle-income nations (2, 11). The recent study discovered similar percentages of overweight throughout quintile 1, 3, and 5 schools, defying previous beliefs that overweight is primarily an urban or high-income phenomenon. This confirms mounting evidence that children from all socioeconomic backgrounds are affected by the nutritional transition, characterized by a decrease in physical activity and an increase in the consumption of calorie-rich, nutrient-poor foods (19).

Particularly in Q5 and Q1 schools, the remarkably high prevalence of overweight and obesity among female students necessitates attention. Similar results showing higher levels of obesity and overweight among females have been noted in South Africa and other nations in sub-Saharan Africa (20). Sociocultural norms, girls’ lower levels of physical activity, and greater exposure to obesogenic dietary environments are all potential contributing factors. The differences in the timing and tempo of the adolescent growth spurt may have influenced the anthropometric outcomes observed in this study. Adolescents who experience earlier maturation often demonstrate increased height, weight, and BMI values at a given chronological age compared to their later-maturing peers, who may appear smaller but subsequently catch up in growth. This variation in growth patterns may partly explain differences in height, weight, and BMI across age groups.

### 4.3 School quintiles, inequality, and nutritional outcomes

The results of the study remain epidemiologically relevant, though there were no significant statistical differences in WAZ, HAZ, and BAZ across the schools. The co-habitation of overnutrition and undernutrition in the country reflects the structural inequality in South Africa, where food access and dietary quality do not consistently correspond with school funding categories (5). School quintile classification is based primarily on community-level socio-economic indicators and funding models, rather than individual household food security or dietary behaviors. Consequently, children in fee-paying schools may still experience inadequate dietary quality, whereas those in no-fee schools may benefit from protection against severe undernutrition through school feeding programs.

### 4.4 Implications for health, education, and policy

The coexistence of undernutrition and overnutrition among school-aged children has a significant impact on their health, academic outcomes, and chronic illnesses (11, 20). Malnutrition has far-reaching effects on school-aged children in the developing world, including their health, academic performance, and access to economic opportunities. A child’s health and academic performance are both negatively affected by undernourishment, which manifests as lowered immunity, increased disease, and stunted growth. Poor academic performance may stem from impaired learning capacity due to cognitive impairments and stunted growth. Educational achievements are affected by undernourishment, which impairs concentration, cognitive capacities, and increases absenteeism due to illness. Inadequate education leads to economic challenges, which, in turn, perpetuate the vicious cycle of poverty. Health issues related to overnutrition also affect school attendance and performance. Childhood obesity increases the risk of developing non-communicable diseases in later life and imposes a significant economic burden on the country’s health system (12). The findings of the study support the relevance and significance of school-based, multi-stakeholder interventions that address both types of malnutrition spectrum. The results support the relevance of the UNICEF-supported Blueprint for Improving the School Food Environment, which emphasizes the availability of healthy food, nutrition education, and supportive school policies (13).

## 5 Strengths

This study demonstrates several key strengths. First, it addresses a significant evidence gap by focusing on children aged 5–13 years, a population frequently underrepresented in national nutrition surveillance. Second, the inclusion of schools from three distinct socio-economic quintiles and both urban and rural environments enables robust comparisons across diverse contexts. Third, the application of standardized WHO anthropometric methods and reference standards strengthens the reliability and comparability of the results. Finally, embedding the study within a broader intervention framework enhances its relevance for policy development and program planning.

## 6 Limitations

Several limitations should be considered when interpreting the findings of this study. The sample size was limited to the number of 5–13-year-old children attending the selected public primary schools whose parents gave voluntary consent. The findings from this sub-study were exploratory and descriptive as it was part of a larger intervention pilot study. Data on birth weight were not collected, which could have provided insight into the reasons for deviations in weight and height scores from the standard population. These differences may indicate previous episodes of malnutrition. This study did not account for differences in the timing and speed of the adolescent growth spurt. These variations can influence height, weight, and BMI at the same chronological age, as early-maturing children often appear larger while later-maturing children may catch up later. Future studies should include measures of biological maturation to better interpret anthropometric findings. Information on dietary intake was also unavailable. Furthermore, body mass index-for-age may not be the most appropriate indicator for this limitation. This limitation may lead to an overestimation of the rates of severe wasting, stunting, and thinness among children. Since BMI provides only a general assessment of nutritional status and does not distinguish between muscle and fat, it has inherent limitations. Determining appropriate cut-off points for nutritional status is also challenging. In this study, cut-off points were set with some degree of arbitrariness, although they accounted for age differences between boys and girls. Multiple methods were employed to assess nutrition, utilizing international reference data to contextualize findings from a developing country. Finally, as only children from public schools were included, the results may not be generalizable to all children in KZN or in South Africa.

Despite these challenges, the study provides valuable new insights into the nutritional status of South African children residing in the iLembe district, an area with limited public health information. These data are essential for formulating urgent intervention strategies. The findings are crucial for developing +effective approaches to address malnutrition-related health risks among children in this region of South Africa.

## 7 Conclusion

This study shows the double burden of malnutrition among primary school children in who participated in the study, irrespective of school quintile or geographic location. The rates of stunting and the high prevalence of overweight and obesity across different school quintiles represent a significant public health problem. This sub-study formed part of a larger study that aimed to determine, implement and evaluate contextualized interventions to improve the school food environment in the selected primary schools in KZN. A school-based, comprehensive nutrition intervention program across schools can support healthy growth and prevent overweight factors that lead to malnutrition. A comprehensive life approach is essential for addressing child health and development, and establishing a healthy school food environment that improves the availability of healthy foods, supports nutrition education, and encourages physical activity is of immense importance in enhancing child health outcomes and achieving both national and global nutrition targets.

## Data Availability

The raw data supporting the conclusions of this article will be made available by the authors, without undue reservation.
